# Sodium glucose co-transport 2 inhibitors for gout treatment

**DOI:** 10.15190/d.2022.11

**Published:** 2022-09-30

**Authors:** Manoj Kumar Reddy Somagutta, Enkhmaa Luvsannyam, Molly Jain, Gaurav Venkat Cuddapah, Sandeep Pelluru, Nafisa Mustafa, Duaa S. Nasereldin, Siva K. Pendyala, Nagendrababu Jarapala, Bhavani Padamati

**Affiliations:** ^1^Department of Family Medicine, Southern Illinois School of Medicine, Springfield, Illinois; ^2^Avalon University School of Medicine, Willemstad, Curacao; ^3^Saint James School of Medicine, Park Ridge, Illinois, USA; ^4^Kamineni Academy of Medical Sciences and Research Center, Hyderabad, India; ^5^The National Ribat University Khartoum, Sudan; ^6^Ahfad University for Women, Omdurman, Sudan; ^7^Atlantic University School of Medicine, Gross Islet, St. Lucia

**Keywords:** Hyperuricemia, Gout, Sodium-glucose transporter-2 inhibitors, Cardiovascular health, Dapagliflozin.

## Abstract

Hyperuricemia remains the most prevalent cause of gout. Gout patients present with joint inflammation and uric acid crystals deposition manifesting as tophi. The association of gout with increased risk of insulin resistance, diabetes, metabolic disorders, increased cardiometabolic risk, and kidney disease is well established. These factors influence the treatment plan, and current treatment options have limited cardiovascular risk reduction. So the need for novel treatments with a broad range of coverage for the complications is warranted. Sodium-glucose co-transporter 2 inhibitors are novel drugs approved for treating type-2 diabetes. They prevent glucose reabsorption and lower serum uric acid levels. Recently few studies have studied their association with reducing the risk of gout. They may help address the gout related complications through their recorded benefit with weight loss, improved insulin resistance, and cardiovascular benefits in recent studies. . SGLT2-Is may be useful to reduce the risk of gout in individuals with type 2 diabetes. Limited literature is available on the safety and efficacy of these novel antidiabetic drugs in patients with gout. This review is aimed to summarize the current knowledge on the role and effectiveness of novel antidiabetic medication as an early therapeutic option in gout patients.

## 1. Introduction

Hyperuricemia is a commonly known clinical syndrome with increased serum uric acid (UA) concentration higher than 7 mg/dl in the body^1^. It is estimated to affect about 8.9% to 24.4% of the general population^2^. It may manifest symptomatically into a cluster of disorders such as gout, urolithiasis, or acute urate nephropathy^3^. It may also be asymptomatic with increased serum UA concentration but no manifestation of symptoms^2,3^. Due to the complexity and controversial treatment options surrounding hyperuricemia, there remains a debate for physician judgment on whether it is important to manage asymptomatic hyperuricemia without any clear signs of danger. However, many studies have reported hyperuricemia as a silent trigger for tissue damage, especially in kidneys^3^. Moreover, increased serum uric acid concentrations have been linked to a variety of comorbidities such as hypertension, dyslipidemia, obesity, metabolic syndrome, type 2 diabetes, cardiovascular disease, and chronic renal disease (CKD)^1,3^.

Gout is the most common presentation of symptomatic hyperuricemia when uric acid crystallization can cause tissue irritation resulting in tissue and joint inflammation with uric acid crystals deposition manifesting as tophi^4^. Lifestyle modifications such as decreased intake of red meat, alcohol (especially beer), and sugary beverages, as well as increased coffee consumption (>4 cups), can help with lowering uric acid uric acid serum levels^3,4^. Weight loss is highly recommended as obesity is linked to hyperuricemia^4^. The first-line pharmacological treatment of hyperuricemia and gout starts with xanthine oxidase inhibitors such as allopurinol and febuxostat^5^. Their mechanism of action hinders the formation of uric acid from uric acid precursors, thereby managing chronic hyperuricemia and lowering serum uric acid concentration^3,4^. They are associated with adverse effects such as rash, pruritus, cytopenia, diarrhea, toxic epidermolysis, and Stevens-Johnson syndrome^3^. Additionally, uricosuric agents such as probenecid could be further added in severe hyperuricemia to aid with faster excretion of uric acid levels^3,5^. However, they are contraindicated in blood dyscrasias such as glucose-6-phosphate dehydrogenase deficiency, uric acid kidney stones and moderate to severe renal impairment. Uricosurics increase uric acid renal excretion by inhibiting URAT1 in renal tubules. Other uricases such as rasburicase or pegloticase (the PEGylated form) are second-line drugs as their usage is limited due to parenteral administration and the risk of infusion reactions^3^. In addition, the association of gout with increased cardiometabolic risk is well established. Using effective and safe treatment for gout is paramount since the randomized CARES (Cardiovascular Safety of Febuxostat and Allopurinol in Participants With Gout and Cardiovascular Comorbidities) trial revealed that febuxostat was associated with a higher risk for cardiovascular death and all-cause mortality compared with allopurinol^6^.

Sodium-glucose cotransporter 2 (SGLT2) inhibitors approved for the treatment of diabetes mellitus have also been reported to lower serum uric acid levels in patients with type 2 DM^7,8^. As per a recent meta-analysis of 15 randomized trials involving 20,241 patients, the SGLT2 inhibitors significantly reduced the all-cause mortality, composite of cardiovascular mortality, heart failure (HF) hospitalizations and urgent visits for HF among patients with HF in all following subgroups: male, female, age < 65, age ≥ 65, race - Black and White, estimated glomerular filtration rate (eGFR) <60, eGFR ≥60, New York Heart Association (NYHA) class II, NYHA ≥III, and HF with preserved ejection fraction^9^. SGLT2 inhibitors improve glycemic levels in type 2 DM by inducing urine glucose excretion due to inhibition of SGLT2 at the S1 segment of the proximal tubule^7,8^. The glycosuria caused by SGLT2 inhibitors helps uric acid be secreted into the urine^7-10^. The most likely mechanism postulated was that, through SGLT2 inhibitors, an increase in uricosuria occurred by suppressing the activity of glucose transporter 9b, a hexose/urate transporter located at the proximal tubular cells across the basolateral membrane^10^. This raises the possibility that lowering uric acid by SGLT2 inhibition may reduce adverse CV events, mainly when used in treating gout, especially when co-existent with diabetes or metabolic derangements. Thus, this review is aimed of summarize the current knowledge on the role and effectiveness of novel antidiabetic medication as an early therapeutic option in gout patients.

## **2. **Hyperuricemia and the cardiometabolic risk

Hyperuricemia pathophysiologically contributes to the development of CVD. It leads to endothelial dysfunction through impaired nitric oxide-mediated vasodilation, increased oxidized low-density lipoproteins, dyslipidemia, and/or acute and chronic inflammation^11^. It is also known that increased SU causes an increase in the levels of C-reactive protein (CRP), tumor necrosis factor, and other systemic markers of inflammation, such as interleukin-1 (IL-1). These factors may contribute to atherosclerosis and endothelial damage leading to CV disease. The endothelial damage and dysfunction may be a pathogenic explanation for an increased incidence of acute vascular events and cardiac events linked with hyperuricemia^11^. In a mendelian randomization study by Kleber and colleagues, each 1-mg/dl increase in genetically predicted uric acid concentration was significantly associated with cardiovascular death and sudden cardiac death^12^. A recent study by the Italian Society of Hypertension which was designed to define the level of uricemia above which the independent risk of CV disease may increase in a significant manner and has shown that, in a cohort of more than 20,000 outpatients, sUA was associated with all-cause and CV mortality, with an optimal sUA cut-off point of 5.6 mg/dL for cardiovascular mortality^13^. Since correlation doesn't equal causation, the causality of the relationship between uric acid and cardiovascular disease remains partly unproven. However, several mechanisms, such as uric acid-induced endothelial dysfunction, oxidative stress, and systemic inflammation, are shared with other CV risk factors, such as hypertension and diabetes, leading to endothelial dysfunction and atherosclerosis and increasing the overall CV risk^13^.

An association between elevated serum uric acid levels and hypertension has been reported in several studies. Kuwabara et al. reported that approximately 25% to 40% of untreated hypertensive patients have concomitant hyperuricemia^14^. In a cross-sectional study conducted by Kuwabara, the analysis showed that an increase in serum uric acid levels by 1 mg/dL increased the prevalence of hypertension by 1.2-fold even when adjusted to age, BMI, dyslipidemia, diabetes, smoking, and estimated glomerular filtration rate^15^. Hyperuricemia also seems to play a role in developing primary hypertension in adolescents. One study identified elevated uric acid levels (>5.5 mg/dL) in nearly 90% of adolescents with essential hypertension^16^. Thus, hyperuricemia needs to be addressed at earlier stages to prevent the risk of primary hypertension. The link between hyperuricemia and the risk of atherosclerosis have been well demonstrated. In a study of young adults, elevated UA is also found to be an independent risk factor for sub-clinical atherosclerosis^17^. In a cross-sectional study of middle-aged adults, high serum uric acid levels were independently associated with the prevalence of atherosclerotic vulnerable carotid plaque^18^.

Gout is also identified as an independent risk of MI. In Krishnan et al.'s study, gout was associated with a higher risk of acute MI (OR 1.26 [P < 0.001]) independent of hyperuricemia^18^. A population-based study found women with gout to have an increased risk for acute MI than those without gout (RR 1.39, 95% CI 1.20–1.61)^19^. A similar finding was noticed in another study where women with gout had a greater risk for all vascular diseases than men (HR, 1.25 vs. 1.06)^20^. Another analysis found that a cohort of patients with gout and no diabetes had a similar risk of stroke but less chance of MI, implying that gout is not a cardiac risk equivalent to MI but is a cardiac risk equivalent to stroke^11,21^.

Hyperuricemia increased the risk for heart failure development and was associated with worse outcomes in a meta-analysis study of 32 studies^22^. In an investigation both a chronic history of gout and an acute gout episode (within 60 days of the event date) were associated with a risen risk of HF readmission or death and continuous allopurinol use for more than 30 days in gout patients did not improve the composite measure of HF readmission and all-cause mortality^23^. In a study by Khan et al., high serum uric acid was observed in 59.29% of patients with CHF, including all classes of NYHA classifications. UA serum levels were reported to be used as a significant prognostic marker in HF patients and could help identify patients at high risk for HF^24^. Another study by Borghi and Colleagues reports increased levels of xanthine oxidase that cause increased production of urate, leading to oxidative stress, thereby causing HF. The study further speculates an association of hyperuricemia to be a poor prognostic marker in HF patients^25^. Hence, acute and chronic gout are associated with deleterious effects in HF patients.

Some studies also noticed the connection of hyperuricemia with type 2 DM and metabolic syndrome. In a study by Woyesa et al.^26^, from 33.8% (n=106) of hyperuricemic T2DM patients, about 27.7% (n=87) of them had metabolic syndrome. This indicates that the prevalence of metabolic syndrome is significantly high among T2DM with abnormal serum uric acid concentrations. There was a positive correlation between high prevalence of metabolic syndrome, 70.1% (n=220) and abnormal serum uric acid concentration, 27.7% (n=87) in T2DM patients (P-value=0.001)^26^. In another study by Arersa et al., hyperuricemia was significantly associated with obesity, duration of DM ≥10 years, having a family history of CVD, alcohol drinking, and increased DBP^27^.

## 3. Role of SGLT2i in hyperuricemia and gout

Uric acid, the prime metabolic intermediate of gout and urate renal stones, is associated with an increased cardiovascular risk burden. Hyperuricemia is an old emerging metabolic disorder, and the interaction between uric acid and cardiovascular diseases has been clearly described. Several illnesses, including hypertension, myocardial infarction, metabolic syndrome, and heart failure, are related to increased uric acid levels^1-3^. Current treatment with traditional UA-lowering drugs has provided conflicting results regarding HF. Even though some report the possibility of preventing HF, the evidence is still lacking if it has a significant clinical benefit. Allopurinol did not improve exercise tolerance in HF in a randomized placebo-controlled trial, despite decreasing B-type natriuretic peptide (BNP) levels^28^. In another study, the use of oxypurinol did not change left ventricular systolic function in an entire cohort of HF patients^29^.

**Figure 1:**
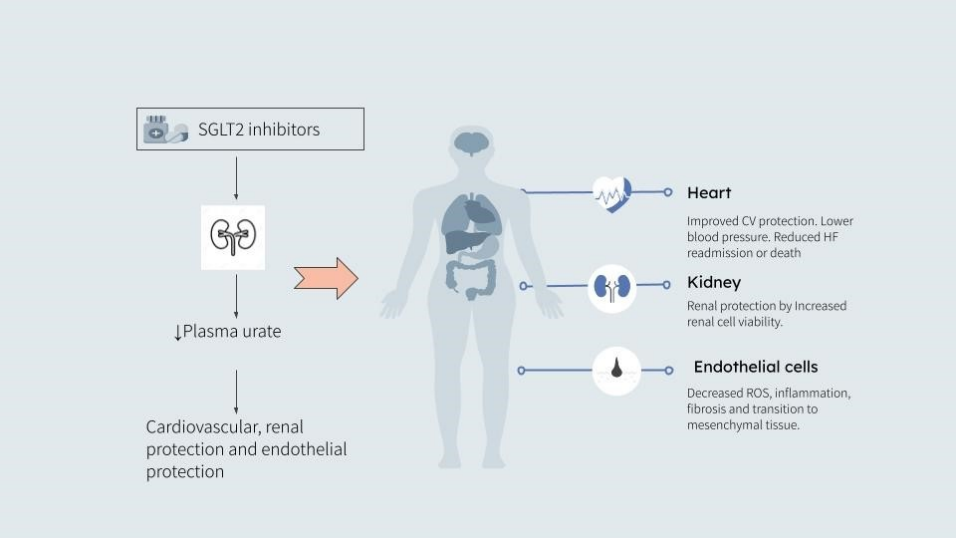
Sodium glucose co-transport 2 inhibitors for gout treatment

Uric acid levels are often elevated in T2DM, contributing to the metabolic syndrome of CV risk. It is hypothesized that treating T2DM with an SGLT2 inhibitor increases UA excretion, reduces circulating UA, and improves parameters of CV and renal function^30^. The probable mechanism is depicted in Figure 1. The more probable mechanism through which SGLT2 inhibitors increase uricosuria and lower circulating uric acid is by suppressing the activity of GLUT9b, which is expressed at the apical membrane of the kidney tubular cells and transports both UA and d-glucose. In 2014, Chino et al. after the oral administration of luseogliflozin, an SGLT2 inhibitor, to healthy subjects, noticed the SUA levels decrease due to an increase in the urinary excretion of UA, which is associated with an upsurge in urinary d-glucose excretion, but not with the plasma SGLT2i concentration^31^. In 2021, a Taiwan nationwide cohort study investigated the association between SGLT2i use for Type 2 Diabetes and the incidence of gout. They analyzed 47,905 individuals receiving an SGLT2 inhibitor and 183,303 receiving a DPP4 47,405 pairs of patients using an SGLT2 inhibitor or DPP4 inhibitor in 1:1 propensity score-matched analyses. The use of SGLT2 inhibitors was associated with a lower risk of gout (HR, 0.89; 95% CI, 0.82-0.96) compared with DPP4 inhibitors, particularly for patients receiving dapagliflozin (HR, 0.86; 95% CI, 0.78-0.95). A sensitivity analysis performed with gout-related medication also showed a significantly lower risk for gout incidence of 15% with SGLT2 inhibitors (HR, 0.85; 95% CI, 0.74-0.97)^32^.

Later that year, a post hoc analysis of pooled data from four placebo-controlled phases III studies assessed the effect of canagliflozin on serum uric acid levels in patients with T2DM and a subset of patients with hyperuricemia^33^. After 26 weeks of treatment, they noticed canagliflozin decreased serum uric acid in patients with T2DM, including those with baseline hyperuricemia, and both canagliflozin 100 and 300 mg dosages were associated with a ∼13% reduction in sUA compared with placebo. They also witnessed that the incidence of gout and kidney stones were low and similar across group^33^. Thus, SGLT2i proved to be the most efficient medication on the market to decrease serum uric acid levels.

In 2018, a meta-analysis of 62 studies, including 34,941 patients, reported that any of the SGLT2 inhibitors (empagliflozin, canagliflozin, dapagli-flozin, tofogliflozin, luseogliflozin, or ipragliflozin) significantly decreased SUA levels compared with control (WMD, -37.73 μmol/L, 95% CI [-40.51, -34.95]). However, treatment with empagliflozin resulted in a superior reduction in SUA compared to others, but there were no consistent dose-related effects^34^. SGLT2i are often used in T2DM and have recently been associated with lower gout rates than glucagon-like peptide-1 receptor agonists (GLP1-RA). A recent Danish study assessed the association between SGLT2-I initiation and gout using a cohort study design comparing the 3-year gout risk among SGLT2-I users with propensity score-matched GLP1-RA users^35^. Among 11,047 pairs of SGLT2-I and GLP1-RA users, the incidence rate of gout was 4.1 and 7.0 events per 1000 person-years, yielding an incident rate difference of 3.0 (95% confidence interval: 4.4 to 1.5) and Hazard ratio of 0.58 (0.44 to 0.75). Hence the initiation of SGLT2-Is was associated with a markedly decreased risk of gout compared to the initiation of GLP1-RAs^35^. A similar population-based cohort study in the U.S by Fralick et al. compared the rate of gout between adults prescribed an SGLT2 inhibitor and those prescribed GLP1 agonists. The study included 295,907 adults with type 2 DM and noticed that the gout incidence rate was lower among patients taking an SGLT2 inhibitor [HR= 0.64 95% CI, 0.57 to 0.72] and a rate difference of −2.9 (95% CI, −3.6 to −2.1) per 1000 person-years. So they observed adults with type 2 diabetes prescribed an SGLT2 inhibitor had a lower rate of gout than those prescribed a GLP1 agonist^36^.

To understand the added benefit of SGLT2i to the traditional UA lowering medications, Stack et al. added dapagliflozin to febuxostat, a xanthine oxidase inhibitor (XOI), and verinurad, a urate transporter 1 (URAT1) inhibitor to assess if it may enhance serum uric acid (sUA) lowering compared to placebo. He noticed dapagliflozin further reduced sUA without influencing uUA excretion, suggesting that its combination with verinurad and febuxostat does not adversely affect kidney function^37^ as summarized in the below Table 1.

**Table 1:** Studies that investigated the effectiveness of SGLT2 inhibitors in reducing serum uric acid Empagliflozin (EMPA), canagliflozin (CANA), dapagliflozin (DAPA), tofogliflozin (TOFO), luseogliflozin (LUSE), urinary excretion rate of uric acid (UEUA).

Study (Country) Objective	Population	Study arm/ Control	Main Outcomes	Others
Prospective case study^31^ (Japan) Mechanism for reduction of sUA and the urinary excretion rate of uric acid (UEUA)	57 and 24 healthy men with a single and multiple dose	Oral administration luseogliflozin/ N/A	- Decreased sUA due to the increase in UA excretion - The increase in UE(UA) correlates with increase urinary D-glucose excretion, but not with the plasma luseogliflozin levels	- Increase in UE(UA) is likely due to glycosuria - Facilitative glucose transporter 9 isoform 2 (GLUT9ΔN, SLC2A9b)
Post-hoc analysis of four placebo-controlled phases III studies^33^ Effect of canagliflozin on serum UA levels in patients with T2DM and in a subset of patients with hyperuricaemia	Patients with T2DM and hyperuricemia	Canaglifloz in 100 and 300 mg for 26 weeks/ Placebo	- Canagliflozin 100 and 300 mg were associated with a ∼13% reduction in serum uric acid compared with placebo.	- Incidences of gout and kidney stones were low and similar across groups
A meta-analysis of randomized controlled trials^34^ (China) To illustrate the effects of SGLT2 inhibitors on SUA in patients with T2DM.	34 941 patients on SGLT2 inhibitors and with T2DM.	Patients on any type of SGLT2 inhibitors for T2DM/ Placebo	- Any of the SGLT2 inhibitors (EMPA, CANA, DAPA, TOFO) significantly decreased SUA levels compared with control	- Empagliflozin resulted in a superior reduction in sUA compared to others. - The effect persisted during long-term treatment
Population-ba sed cohort study^35^ (Denmark) Assess the association between SGLT2-I initiation and gout	Comparing the 3-year risk of gout among SGLT2-i users vs. GLP1-RA users.	Patients initiated on SGLT2-inhibitors	- Patients initiated on GLP agonists	- Gout incidence rate was lower among SGLT2 Inhibitor users than GLP1 agonists
Population-ba sed new-user cohort study^36^ (USA) Assess the association between SGLT2-I initiation and gout	T2DM newly prescribed an SGLT2 inhibitor were matched to patients newly prescribed a GLP1 agonist.	Patients newly started on SGLT2-inhi bitors	- Patients newly started on GLP agonists	- Gout incidence rate was lower among SGLT2 Inhibitor users than GLP1 agonists
Randomized, placebo-controlled, 2-way crossover study^37^ (USA) To assess whether dapagliflozin added to verinurad, a selective URAT1 inhibitor, and febuxostat, an XOI, increases uUA excretion.	Adults with asymptomatic hyperuricemia.	Patients initiated on SGLT2-inhibitors	Patients initiated on GLP agonists	- Gout incidence rate was lower among SGLT2 Inhibitor users than GLP1 agonists

A recent meta-analysis by Bhattarai et al. assessed the CV outcomes associated with treating patients with SGLT2i by analyzing the combined data from 10 RCTs. Significantly, a lower number of patients treated with SGLT2i experienced a primary outcome event (defined as the CV death and hospitalization for HF) with OR, 0.67 [95% CI, 0.55-0.80, P < .001]^38^.

SGLT2 inhibitors may lower a person’s risk for gout but are associated with several adverse events. The most common adverse event is a genital infection, affecting approximately 7% of patients who receive an SGLT2 inhibitor^39^. Female sex and history of prior infection are the components that increase the risk of genital infections with SGLT2i therapy^40^. However, most genital infections following SGLT2 inhibitor therapy are mild to moderate and respond well to conventional treatment. Other rare adverse events include euglycemic diabetic ketoacidosis and lower-limb amputation^35,41^. In a recent meta-analysis comparing patients using SGLT2i with non-SGLT2i users, amputation risks and peripheral arterial disease (PAD) slightly increased in patients with canagliflozin treatment (amputation: OR=1.60; PAD: OR=1.53)^42^. Lower baseline diastolic blood pressure and more systolic blood pressure reduction significantly increased risks of amputation and PAD, respectively, in patients with SGLT2i treatment^42^. On top of their effectiveness, most economic evaluations of SGLT2 inhibitors are considered economical and cost-effective compared to insulin, thiazolidinediones, sulfonylureas, and GLP1 agonists in diabetic patients^43,44^.

## 4. Conclusion

Hyperuricemia is associated with an increased risk of cardiovascular disease, type 2 DM, metabolic syndrome, gout, and various severe manifestations.

Treatment of hyperuricemia and gout focuses on lifestyle modifications and different pharmacological agents such as XO inhibitors, uricosurics, and urate transporter inhibitors.

However, this treatment strategy does not successfully address the long-term cardiometabolic consequences of hyperuricemia. There is yet some ambiguity when deciding on a therapeutic agent that can treat hyperuricemia and manage the associated comorbidities. SGLT-2 inhibitors, due to their effectiveness in controlling UA levels and type 2 DM and improving cardiovascular health and lipid profile, arise as a potential new treatment option for treating gout.

## KEY POINTS

**◊** *Gout is the most common presentation of symptomatic hyperuricemia, being associated with increased cardio metabolic risk.*

**◊** *Increased serum uric acid correlates with systemic markers of inflammation, contributing to atherosclerosis and endothelial damage*

**◊** *SGLT2 inhibitors have also been reported to lower serum uric acid levels in diabetic patients*

**◊** *Treatment with an SGLT2 inhibitor increases uric acid excretion, reduces circulating uric acid, and improves cardiovascular and renal parameters*

**◊** *SGLT-2* *inhibitors arise as a potential new treatment option for treating gout*
